# Rating analysis and BERTopic modeling of consumer versus regulated mHealth app reviews in Germany

**DOI:** 10.1038/s41746-023-00862-3

**Published:** 2023-06-21

**Authors:** Marie Uncovska, Bettina Freitag, Sven Meister, Leonard Fehring

**Affiliations:** 1 Faculty of Health, School of Medicine, Witten/Herdecke University, Witten, Germany; 2 Health Care Informatics, Faculty of Health, School of Medicine, Witten/Herdecke University, Witten, Germany; 3 Department Healthcare, Fraunhofer Institute for Software and Systems Engineering, Dortmund, Germany; 4 Gastroenterology, HELIOS University Hospital Wuppertal, University Witten/Herdecke, Wuppertal, Germany

**Keywords:** Health policy, Health care economics

## Abstract

Germany introduced prescription-based mobile health (mHealth) apps in October 2020, becoming the first country to offer them fully reimbursed by health insurance. These regulated apps, known as DiGAs, undergo a rigorous approval process similar to pharmaceuticals, including data protection measures and sometimes clinical trials. This study compares the user experience of DiGAs with non-prescription mHealth apps in Germany, analyzing both average app store ratings and written reviews. Our study pioneers the use of BERTopic for sentiment analysis and topic modeling in the mHealth research domain. The dataset comprises 15 DiGAs and 50 comparable apps, totaling 17,588 German-language reviews. Results reveal that DiGAs receive higher contemporary ratings than non-regulated apps (Android: 3.82 vs. 3.77; iOS: 3.78 vs. 3.53; *p* < 0.01; non-parametric Mann–Whitney–Wilcoxon test). Key factors contributing to positive user experience with DiGAs are customer service and personalization (15%) and ease of use (13%). However, challenges for DiGAs include software bugs (24%) and a cumbersome registration process (20%). Negative user reviews highlight concerns about therapy effectiveness (11%). Excessive pricing is the main concern for the non-regulated group (27%). Data privacy and security receive limited attention from users (DiGAs: 0.5%; comparators: 2%). In conclusion, DiGAs are generally perceived positively based on ratings and sentiment analysis of reviews. However, addressing pricing concerns in the non-regulated mHealth sector is crucial. Integrating user experience evaluation into the review process could improve adherence and health outcomes.

## Introduction

Mobile health (mHealth) applications (apps) have experienced a boom in the past decade, with a global market valuation of 56.8 billion USD in 2022 and more than 350,000 mHealth apps available in app stores^1^. For a long time, they operated in a legal gray area—not classified as medical devices, in most countries they were subject to few regulations, skirting the necessity to provide clinical data to back up health claims^2,3^. In an effort to mitigate some of the safety and security issues facing mHealth apps, several governing bodies have begun to take action: the US Food and Drug Administration (FDA) piloted the introduction of a “precertification” program for mHealth apps^2^, the National Health System (NHS) in the UK launched an “NHS app library”^4^, and Germany introduced the Digital Healthcare Act (“Digitale-Versorgung-Gesetz”, DVG)^5,6^, in 2020, taking a pioneering role worldwide in implementing mHealth into the standard of care. The DVG and subsequent legislation (such as the DiGAV^7^) entitle statutorily insured patients to receive fully reimbursed prescription mHealth apps called DiGAs (“Digitale Gesundheitsanwendungen”) and establish a comprehensive certification process for such apps that requires scientific proof of effectiveness through a comparative study. Other certification requirements include safety, functionality, quality, interoperability, data protection, and data security. This differentiates these regulated apps from consumer mHealth apps, which are not subject to the same level of regulatory scrutiny and are typically marketed directly to consumers. There are two types of DiGAs: provisionally listed and permanently listed. Provisionally listed DiGAs are evaluated as potentially beneficial by the BfArM (German Federal Institute for Drugs and Medical Devices, “Bundesinstitut für Arzneimittel und Medizinprodukte”) and provisionally listed for 12 months. Manufacturers must provide additional data within this period to prove effectiveness, safety, and economic benefits. If insufficient evidence is provided, the app is removed. Permanently listed DiGAs meet all requirements and are evaluated as effective, safe, and cost-effective. These apps are listed indefinitely, subject to ongoing monitoring by BfArM.

The German system with prescription mHealth apps is, to date, unique worldwide and offers great potential for research centered around mHealth adoption, user experience, and acceptance, especially with regard to prescription and reimbursement status. Past research analyzed user-perceived discrimination of regulated and consumer mHealth apps based on the joint evaluation of user comments and app downloads, finding that the existence of a quality mark alone is not a significant or relevant factor in user experience^8^. Nonetheless, 2 years after the introduction of the German laws, there is little research on the relationship between reimbursement and prescription status and user perception and experience. Though a significant body of research focuses on a qualitative, clinical evaluation of mHealth apps in Germany, using e.g., the mobile app rating scales (MARS)^9–14^ it centers around physician’s/healthcare practitioners’ perspectives of mHealth apps, thus disregarding the users’ point of view, and does not contrast DiGAs against “regular” mHealth apps. Thus, the aim of this paper is to evaluate the relationship between prescription and reimbursement status and user experience and contrast it against mHealth applications without reimbursement and prescription status. Results reveal that despite having lower aggregate star ratings, DiGAs receive higher contemporary ratings than non-regulated apps (Android: 3.82 vs. 3.77; iOS: 3.78 vs. 3.53; *p* < 0.01; non-parametric Mann–Whitney–Wilcoxon test). Key factors contributing to positive user experience with DiGAs are customer service and personalization (15%) and ease of use (13%). However, challenges for DiGAs include bugs (24%) and a cumbersome registration process (20%). Negative user reviews highlight concerns about therapy effectiveness (11%). Excessive pricing is the main concern for the non-regulated group (27%), while data privacy and security receive limited attention from users in both groups (DiGAs: 0.5%; comparators: 2%). In conclusion, DiGAs are generally perceived positively based on ratings and sentiment analysis of reviews. However, addressing pricing concerns in the non-regulated mHealth sector is crucial. Integrating user experience evaluation into the review process could improve adherence and health outcomes.

## Results

### Comparing differences between DiGAs and comparators—average user star ratings

The overall star rating is only available in aggregate form; therefore, the below analysis is not limited to the period in which DiGAs were on the market but considers the entire period since an app’s first listing. Nonetheless, the overall star rating is often the first information a potential user sees and serves as a key decision criterion to download or not to download an app^15^.

On average, the DiGAs in our sample have 439 total ratings across platforms, while comparators, being on the market longer, have 61,085. Indications with the highest average user ratings include obesity, insomnia, and social phobias/panic disorders, while cancer and impotence apps have the lowest average number of ratings. Apps on the Android platform have 5.5 times as many ratings as on iOS. 75% of selected apps do not have a consistent star rating across platforms. Apps on iOS are rated 0.19 stars higher on average (median = 0.3 stars higher); this effect is amplified when computing the AUR. For a more detailed description of the general data structure and secondary findings, see Supplementary Notes and Supplementary Table 1.

**Fig. 1:**
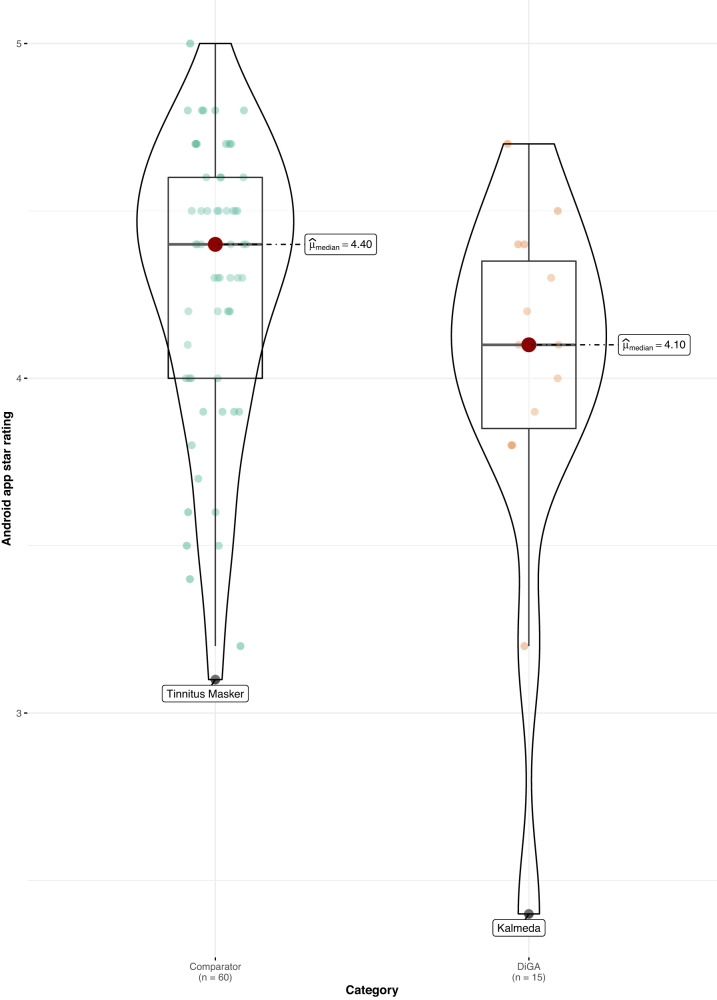
User star rating distribution across groups and platforms (Android). Corresponding test statistics (Mann–Whitney test): W = 602, *p* = 0.04, *r*(biserial) = 0.34, CI (95%) = [0.02, 0.59], *n* = 75. Green dots = aggregate app star rating for the comparator group; one dot represents one app. Orange dots = aggregate app star rating for the DiGA group; one dot represents one app. Black line with red dot = median aggregate app star rating. Boxviolin plot = distribution of aggregate app star ratings. Bounds of box show the lower and upper quartiles (Q1 and Q3). Whiskers show min–max.

**Fig. 2:**
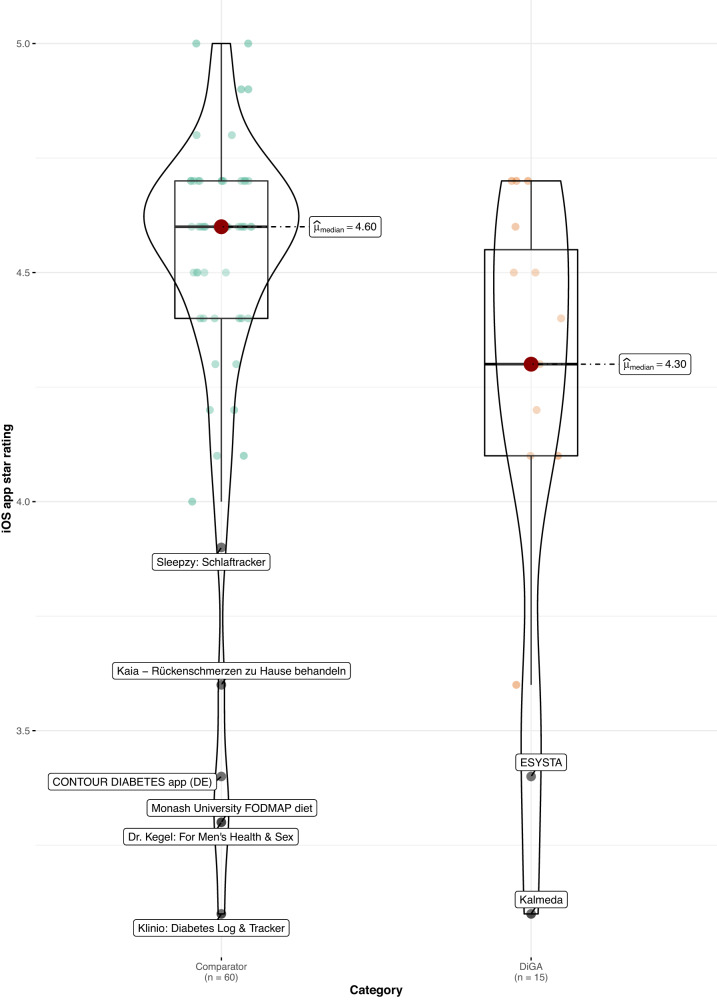
User star rating distribution across groups and platforms (iOS). Corresponding test statistics (Mann–Whitney test): W = 605.50, *p* = 0.04, *r*(biserial) = 0.35, CI (95%) = [0.03, 0.60], *n* = 75. Green dots = aggregate app star rating for the comparator group; one dot represents one app. Orange dots = aggregate app star rating for the DiGA group; one dot represents one app. Black line with red dot = median aggregate app star rating. Boxviolin plot = distribution of aggregate app star ratings. Bounds of box show the lower and upper quartiles (Q1 and Q3). Whiskers show min–max.

**Fig. 3:**
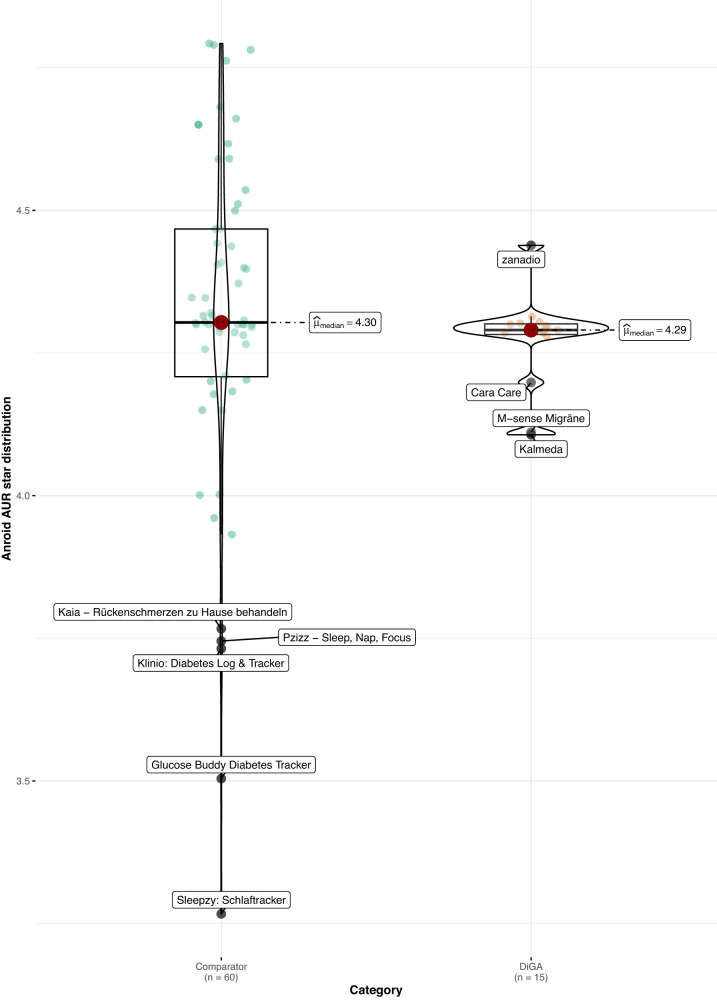
Aggregate user-perceived star rating distribution across groups and platforms (AUR) (Android). Corresponding test statistics (Mann–Whitney test): W = 562, *p* = 0.14, *r*(biserial) = 0.25, CI (95%) = [−0.07, 0.52], *n* = 75. Green dots = aggregate app star rating for the comparator group; one dot represents one app. Orange dots = aggregate app star rating for the DiGA group; one dot represents one app. Black line with red dot = median aggregate app star rating. Boxviolin plot = distribution of aggregate app star ratings. Bounds of box show the lower and upper quartiles (Q1 and Q3). Whiskers show min–max.

**Fig. 4:**
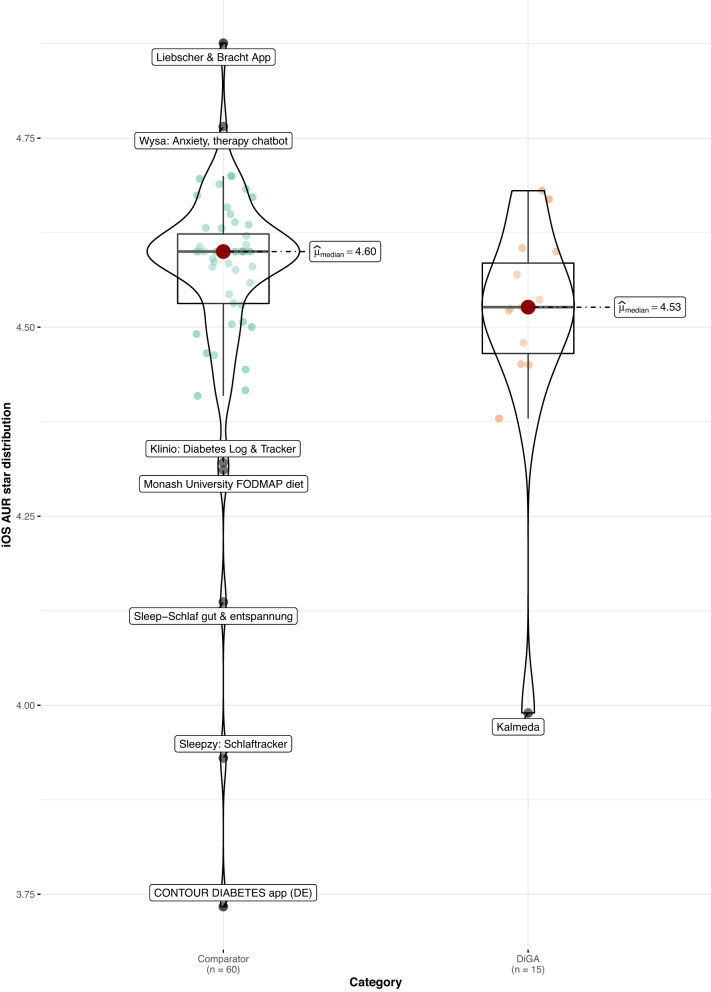
Aggregate user-perceived star rating distribution across groups and platforms (AUR) (iOS). Corresponding test statistics (Mann–Whitney test): W = 600, *p* = 0.05, *r*(biserial) = 0.33, CI (95%) = [0.02, 0.59], *n* = 75. Green dots = aggregate app star rating for the comparator group; one dot represents one app. Orange dots = aggregate app star rating for the DiGA group; one dot represents one app. Black line with red dot = median aggregate app star rating. Boxviolin plot = distribution of aggregate app star ratings. Bounds of box show the lower and upper quartiles (Q1 and Q3). Whiskers show min–max.

When comparing DiGAs to the consumer mHealth apps, average user ratings are worse in both app stores (Android: 4.1 vs. 4.4 stars, *p* < 0.05; *z* = −2.02; *r* = 0.24; size of effect as determined by Cohen^16^ = small; iOS: 4.3 vs. 4.6 stars, *p* < 0.05; *z* = −2.08; *r* = 0.24; size of effect = small; non-parametric Mann–Whitney–Wilcoxon test). Using the non-parametric Mann–Whitney–Wilcoxon test to compare rating distribution, we can confirm that the differences in user star rating distributions between groups are significant; as Levene’s test confirms the homoscedasticity assumption, the difference in medians is also significant (see Figs. 1 and 2). The same holds when calculating the AUR, however, the differences in distributions and medians are no longer significant for both app stores, but only for the iOS platform (*p* < 0.05; *z* = −1.99; *r* = 0.23; size of effect = small; non-parametric Mann–Whitney–Wilcoxon test), as can be seen in Figs. 3 and 4.

However, considering only reviews since the introduction of DiGAs, user star ratings are on average more positive for DiGAs. As a group, DiGAs receive higher star ratings than the comparators on both platforms within this timeframe (on Android: 3.82 vs. 3.77; on iOS: 3.78 vs. 3.53; the difference is significant at *p* < 0.01; non-parametric Mann–Whitney–Wilcoxon test). Thus, the aggregate star ratings mask the fact that when only contemporaneous ratings are considered, regulated apps outperform the comparator group of non-regulated consumer apps.

### Comparing user experience as evaluated by main topics and sentiment analysis of written reviews

The next set of analyses is based on textual user-generated app reviews. To ensure comparability of the information, only textual reviews from the first date of entry of the relevant indication into the DiGA directory until August 4, 2022, are included in the analysis.

For the topic model, in each category, a proportion of reviews focuses on content very specific to the individual therapy areas. As the reviews allocated to this category contain a mixture of topics (ranging from laudatory talk to ease of use to therapy effectiveness), this category (“Therapy area specific”) yields little informational content for comparison; it was however kept for transparency purposes.

**Fig. 5:**
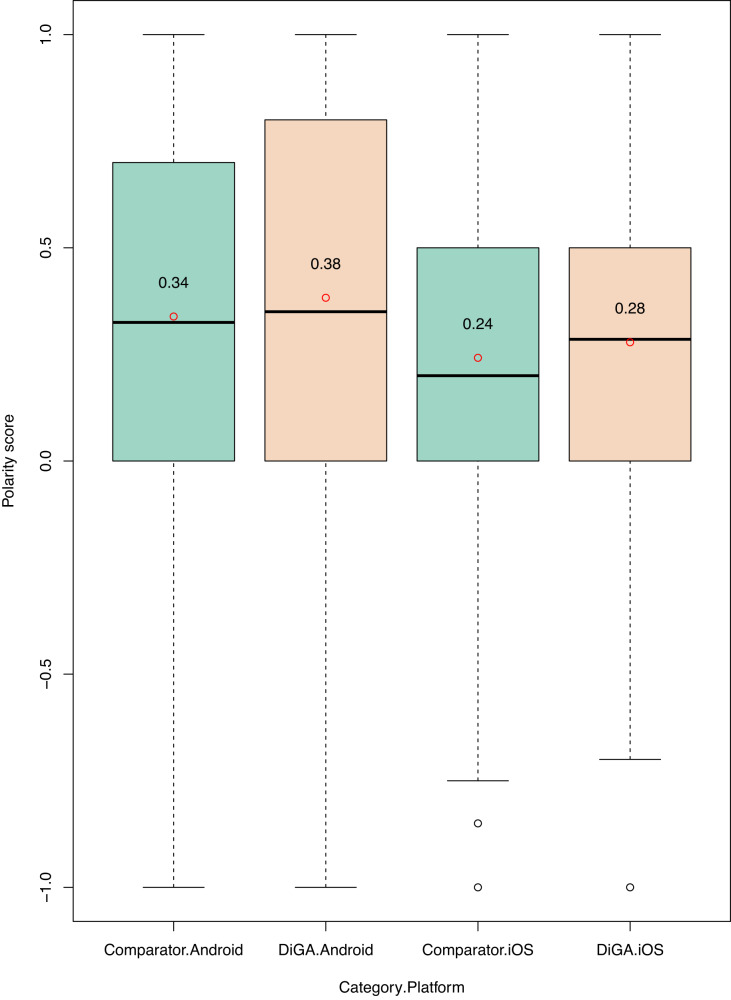
Sentiment analysis—polarity score distribution across groups and platforms. Green boxplot = distribution of polarity scores for comparator group. Orange boxplot = distribution of polarity scores for the DiGA group. Bounds of box show the lower and upper quartiles (Q1 and Q3). Whiskers (dotted line) show min–max. Black line with red dot = median sentiment score.

We next assess the sentiment of textual reviews and analyze differences between groups. In line with the numeric star ratings of the textual reviews from October 2020 onward, the sentiment of textual reviews is on average more positive for DiGA reviews, across platforms (see Fig. 5). These differences are significant for both platforms at a *p* < 0.01 level (non-parametric Mann–Whitney–Wilcoxon test).

Given that app review texts are very short, prone to spelling errors and slang, and often rely on emoticons to transfer meaning, sentiment analysis is often imprecise, achieving accuracy rates of around 70%^17^.

Thus, to validate these first results, we conducted a second analysis using the German sentiment classifier with BERT. This classifier assigns reviews to three categories: negative, neutral, and positive. Comparing the results to the textblob analysis above, we can confirm our findings: on the Android platform, reviews for DiGAs are classified as “positive” 64% of the time, while for comparators only in 61% of cases; on iOS, the discrepancy is slightly higher at 62% of DiGA reviews and 52% of comparator reviews.

**Fig. 6:**
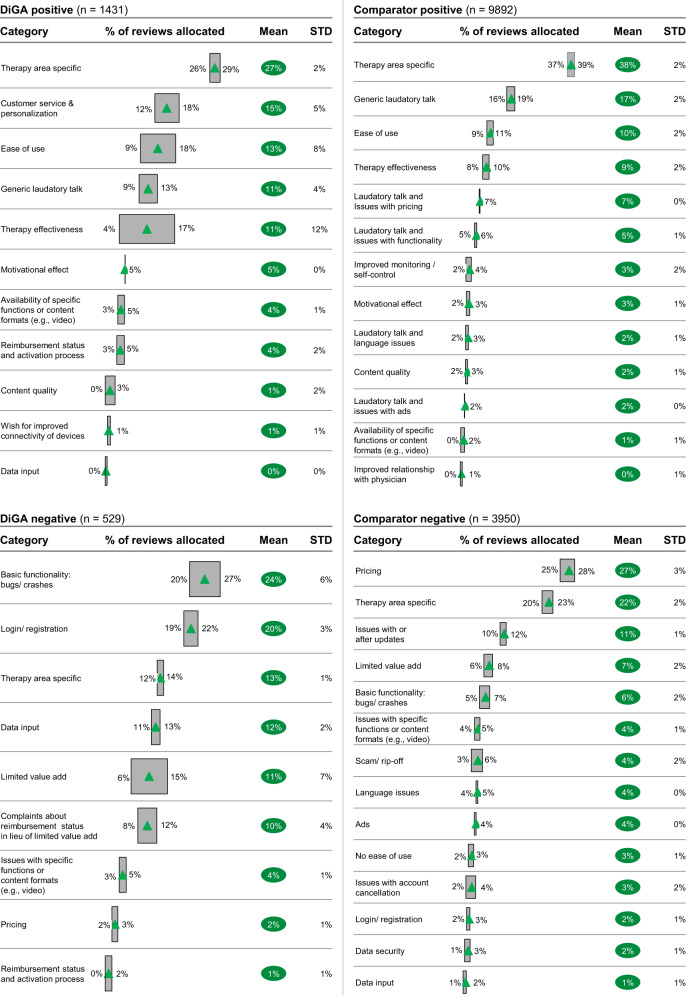
Topic modeling—allocation of reviews to categories. Green triangle = mean % of reviews allocated to category. Gray box = upper and lower bound of 95% confidence interval of % of reviews allocated to category. Green bubble = mean % of reviews allocated to category. STD standard deviation.

Analyzing the mean number of reviews allocated to each category by the topic model (see Fig. 6) and leaving aside therapy area-specific comments, positive reviews for DiGAs most frequently contain praise for great customer service and personalization options (15%). This category is completely missing for the comparator group. One of the DiGA users describes their experience as follows (all example reviews were translated from the German language original only for demonstration purposes; analyses were conducted with original German language text):

“Easy to understand, helpful recommendations, easy to implement. Friendly and competent support from a great team of coaches. This makes losing weight fun!”

Ease of use was a topic reviewers commonly complimented among both the DiGAs and comparator groups, with 13% of positive reviews for DiGAs and 10% of positive reviews for comparators mentioning it. Similarly, therapy effectiveness was also discussed at roughly the same frequency across both groups (11% of DiGA and 9% of comparator positive reviews). One user describes their experience with a DiGA as follows: *“Clear, versatile, comprehensive, easy to use”*, another highlights *“Very good app. Extremely helpful. I am finally making progress with my nutrition!”*

A reviewer using one of the comparator mHealth apps states: “*Very helpful app. Even the free version can already make a lot of difference and contains some useful features. Recommend!*”

Despite having a positive star rating of 4 or 5 stars, the comparator group has a high share of comments that include a wish for improvement or criticism about the app, such as issues with pricing (7%), issues with functionality (5%), or language issues due to the app only being available in English or the German language content being of bad quality (2%). Several illustrative examples of user reviews that align with this classification include, for example,

*“I like it, but the price is unfortunately too high for me”, “It’s quite nice, if the full version were more affordable I would get it, but I feel 7 euros quite expensive for the fact that it only calculates a few things and includes a few minigames”, “Almost everything is great - now it would be fantastic if all content was available in German.”* Such reviews are rarer for DiGAs and center around the wish for improved connectivity of devices (such as trackers, Fitbit, etc.; 1% of comments).

Looking into negative (1- or 2-star-rated reviews), DiGA users most often complain about bugs and crashes, and issues with login/registration (24% and 20% of negative DiGA reviews, respectively).

*“Unfortunately, can’t register at all…?”, “A great idea but poorly implemented. For WEEKS the app has been stuck. This has not happened with any other app before. And whenever I want to input something, I have to wait forever until the app updates itself and saves the things that I enter.”*

This type of comments is less common in the comparator group, where only a smaller share of reviews can be allocated to issues with functionality (11% of negative reviews mention issues with or after updates and only 6% revolve around bugs or crashes).

In the comparator group, 27% of negative reviews is centered around excessive pricing. A clear theme also emerges around scam/rip-off tactics, with 4% of negative comparator reviews warning other users about the app and another 3% experiencing issues with the cancellation of their (paid) subscription. To highlight some user experiences:

*“The app is just insanely expensive.”, “Attention rip-off! Took me half an hour to cancel within the trial period. It is VERY complicated. Many give up for sure and pay the 50€ for a bad app.”, “My subscription was simply extended, and money debited although I have already canceled all subscription on the day of purchase!”*

Complaints about pricing are far less common in the DiGA group (2% of negative reviews). An additional keyword search reveals that the words “rip-off” or “scam” are mentioned in only 1.9% of negative DiGA reviews (10/529) compared to 7.2% of negative comparator reviews (286/3950).

For DiGAs, limited value add is thematized in 21% of negative reviews: 11% of negative reviews center around the lack of value add through using the app, while an additional 10% focuses on complaints about reimbursement status in lieu of limited value add. Some exemplary comments include, for example,

*“Over 200€ for one quarter? Even if the health insurance company covers the cost I consider this pricing exorbitant. I would definitely refuse to use it for this price. No matter who pays.”, “It looks nice, but has a lot of teething problems that make daily use unnecessarily cumbersome. In terms of content, it is limited to simply commanding you not to go to bed until extremely late (1:30). However, it leaves you alone with the implementation. That would have been the essential point.”, “Technically unfinished and amateurish. Rudimentary functions, for me it is puzzling how it could gain medical approval!”*

For the comparator group, the share of reviews addressing specifically a low value add is lower at only 7% of reviews.

It is interesting to note that data privacy and data security only play a minor role in user reviews. The reviews for DiGA mention data security in less than 0.5% of cases (the keywords “data security” or “data protection” only appear in three reviews overall), in the comparator group only 2% of reviews center around this topic.

## Discussion

The significance of reviews and ratings and their association with mHealth outcomes and patient experience is disputed in mHealth research^18^. Nevertheless, ratings serve as a vital decision criterion for prospective users to download an app as they are usually the first information encountered^19^. Despite a wealth of literature on mHealth apps in general and user-generated app reviews in particular, research on the relationship between prescription and reimbursement and user experience is still in its infancy. A significant body of research focuses on a qualitative, clinical evaluation of mHealth apps in Germany, centering around physician’s/healthcare practitioners’ perspectives of mHealth apps^9–12^, whereas international publications have focused predominantly on app development issues^20,21^, and feature evaluation^22–24^ to make mHealth apps more accessible and user-friendly. While certification has been a theme researched in the context of mHealth apps^8^, there simply has been no real-world data comparing the user experience with regulated mHealth apps to that with their consumer counterparts, as such apps did not exist. As such, our research provides invaluable insights into user experience with mHealth under the unique German system of prescription and full reimbursement. Additionally, our study pioneers the application of BERTopic, a novel topic modeling method, in mHealth and provides proof-of-concept for its viability and usefulness in this research domain.

The difference in cross-platform ratings and rating numbers can only partially be explained by higher market penetration rates of Android smartphones when compared to iOS in Germany: according to an aggregated sales figures report^25^, Android phones had a 72.8% market share, compared to a 27.1% share for iOS phones. Previous research comes to similar results: another study found that when comparing cross-platform app pairs, in 63% of cases, Android versions have more users rating them^20^.

The discrepancy in the number of reviews by indication can be explained by market maturity, as indications with a high number of reviews (such as obesity) have been on the market longer than indications with a lower number of reviews (e.g., impotence, cancer).

When looking into the discrepancy in hybrid app ratings between platforms, our results are in line with previous research, which finds that hybrid apps have inconsistent ratings across platforms^20,21^.

It is true that when looking into the aggregate star rating (independent of date), DiGAs perform significantly worse than the comparator group as measured by average user-perceived star rating. This could be explained by three factors: first, when apps initially come to market, they tend to be buggier and face more scrutiny^26^. A study on the evolution of user-generated ratings by app category found that 58% of medical apps in particular tend to improve their star rating after an initial 12-month period^27^. This is also in line with the findings from our topic model: 27% of negative reviews for DiGAs center around basic functionality and bugs.

The aggregate star rating, comprising all reviews regardless of the publication date, serves as a preliminary indicator of an app’s subjective quality for users. However, in examining the relationship between prescription and reimbursement status and user experience as reflected in app store ratings, this metric poses challenges. The DiGA system was only introduced in late 2020, meaning prior reviews of the comparator apps were written by users who could not utilize the prescribed and reimbursed DiGAs. Thus, for the purposes of our study, the star ratings that were published after the introduction of the DiGA system hold greater relevance. Considering this metric, DiGAs perform significantly better in terms of aggregate user-perceived star ratings than the comparator group. This higher star rating and generally more positive sentiment of user reviews for DiGAs can be explained by two factors: as previous research has found, after reaching a critical threshold, higher numbers of ratings tend to encourage negative user star ratings and discourage extremely positive ratings, leading to an overall negative trend in posted ratings over long timeframes^28^. In our case, most of the comparators are longer on the market, better established and thus have a higher number of ratings, while DiGAs represent the new entrants with fewer average ratings. A second trend that could play into higher average recent star ratings for DiGAs is the fact that these apps are more innovative, offer a better service, or have a better-perceived quality than the more established comparators—all while being free for the user, which gives them a leg up when compared to other mHealth applications. Given that the comparison of recent reviews after the introduction of DiGAs offers a more accurate representation of user preferences, it is reasonable to conclude that users generally favor DiGAs over comparators.

The strong focus on customer service and personalization among positive DiGA reviews could be driven by two things: first, this type of intervention is still new to patients in Germany, requiring a higher level of support; second, DiGA users tend to be, on average, 45 years old or older and therefore older than the average mHealth app user in Germany^29,30^, implying a higher emphasis placed on customer service. Previous research finds that personalized approaches to mHealth render stronger behavior changes^31^. Providers of DiGAs should thus not only further build on their customer service to drive adoption rates, but moreover focus on the app’s personalization features to enhance the medical benefit to patients.

The fact that 31% of negative reviews in the comparator group pertain to high prices and scam tactics is not surprising. The mHealth market is intransparent, with patients often lacking the adequate knowledge and tools to identify which apps are right for them personally, but also to judge the adequate price for and the medical benefit of an individual mHealth application^3^. Several cases gained negative publicity in the past, with apps misleading patients with unsubstantiated claims^32–34^ A large-scale mHealth user review study^35^, which analyzed 5 million reviews from nearly 300 apps, found that 44% of the patient reviews mentioning payments gave the app a 1- or 2-star rating. Helping patients navigate this intransparent marketplace through a comprehensive certification process and making mHealth accessible for everyone by reimbursing them is at the very core of the value proposition of DiGAs.

It is interesting to note that the share of reviews complimenting ease of use and therapy effectiveness does not differ between the DiGA and comparator groups. Our hypothesis that DiGAs would exhibit lower ease of use as a result of the prioritization of clinical evidence over user experience by DiGA providers was not supported. This discrepancy may indicate that our assumption was incorrect, or it could be due to a decreased emphasis on ease of use among users. A previous survey on acceptance criteria for mHealth in Germany^30^ found that ease of use was not a significant predictor of intention to use mHealth, indicating that fewer users may be commenting on or perceiving differences in ease of use due to it being of lesser importance to them. However, the same study found that therapy effectiveness was a major predictor of intention to use. This raises concerns as in spite of the comprehensive certification process and the requirement for demonstrating clinical evidence, positive user comments on therapy effectiveness are equally frequent between DiGAs and the comparator group and 11% of negative DiGA reviews criticize limited value add. This suggests that the certification process and emphasis on clinical evidence may not be effectively translating into perceived therapy effectiveness among users.

DiGA providers could improve patients’ understanding of therapy effectiveness by actively seeking feedback from users, regularly updating the user experience, and using analytics on user-generated data to gain insights into the main issues. Targeted information campaigns for both users and healthcare professionals may also help solve the problem. A study among 1308 German healthcare professionals found that only 30.3% would prescribe a DiGA^36^, pointing toward high skepticism among this stakeholder group; a recent report published by a statutory health insurance indicated that only 4% of German physicians had actually prescribed a DiGA to date^29^. As most patients (89%)^37^ get their DiGA prescription directly from their physician, their cautious attitude could affect patient expectations. Here, it was a step in the right direction for the German statutory insurances offering reimbursement for physicians’ time spent on prescribing and monitoring mHealth apps instead of just the reimbursement of the prescription itself^19^, as this increases the time available to physicians to explain the app, manage expectations and ensure users receive the support needed to manage their own health.

It stands within reason that the main issues users address within negative DiGA comments revolve around basic functionality and the registration process. DiGAs are a relatively new technology and are still prone to bugs. However, it is imperative that the providers of DiGAs address these issues as soon as possible, as these are medical products that individuals depend upon for the management of their health. In particular, the registration process can be perceived as cumbersome, and there might be room for optimization in streamlining this process to make it more user-friendly. One of the most commonly mentioned issues pertaining to registration was the repetitive input of data—this problem could easily be alleviated by making data input easier (e.g., by offering to autocomplete suggestions, using voice recognition for data input, and allowing users to save their progress and return to complete later) or using digital identity technologies (e.g., connected to biometric authentication or the insurance ID, connected to electronic health records to transfer data) to reduce the number of data points users need to manually enter.

Physicians are an important link between patients and DiGAs. However, it is clear that their level of knowledge is an obstacle to widespread prescribing^38^. In the future, therefore, the effects of digital literacy among physicians and patients must also be considered. A lack of competence influences acceptance due to a reduced sense of well-being and thus also reduced motivation, according to the Self-Determination Theory^18^.

It is surprising to note that the role of data privacy and security in mHealth has received limited attention in the reviews, despite its prominent position in public discourse in Germany. This could be driven by selection bias (only individuals who use the app leave reviews; this excludes individuals worried about data protection issues, as they might choose to simply not use the app). Alternatively, it could be that users are less concerned about data security and privacy than the public discourse suggests: the previously mentioned survey study among 1349 German adults^30^ found that not only did data security and privacy concerns not affect the respondents’ intention to use mHealth, but also that only 15% of respondents expressed disagreement to the statement “mHealth apps/ DiGAs ensure that the data collected is used only for its intended purpose”. Moreover, a survey of 1003 German adults during the Covid-19 pandemic showed a high willingness to share health data for research^39^. DiGAs already are subject to some of the strongest regulations globally under the General Data Protection Regulation (GDPR), which could additionally lead to users expressing less concern about this issue. For regulators and providers alike, this implies that while data protection is important, it clearly is not the primary driver of user behavior and satisfaction in the context of mHealth. Nonetheless, with ample room to misuse lax security measures, it is important for regulators to keep GDPR in mind when introducing mHealth initiatives.

There are several limitations to our study design. First, despite selecting the comparator group carefully among the cross-platform apps within the same therapeutic area as the DiGAs, the choice of keywords or selection criteria might have led to a skew in our results. Given the sheer number of available apps, we limited content analyses to representative apps from each therapeutic area and only those submitted in German, and such reviews may not accurately represent the entire population of app users. Second, as typical with studies relying on user-generated content, the reliance on subjective data may lead to bias in the results, as star ratings and review content may not accurately reflect the true effectiveness of the app. Despite pre-processing the dataset, data quality may be inadequate, as poorly written or fake reviews may compromise the validity of the findings. Additionally, selection bias may be present, as the app users who leave reviews may not be representative of the general population who use the app. Furthermore, the results may not be generalizable to other populations, as the findings are specific to the population that used the app. The inclusion of non-CE-certified mHealth apps in the comparator group might be another source of bias, as these apps could address a different user group. However, this comparison provides a real-world perspective on how patients use these apps interchangeably to manage their health—a recent survey study found that patients differentiate little between DiGAs and non-regulated mHealth apps^30^. A user-centered approach is crucial for evaluating the effectiveness of the apps, and users often choose apps based on their perceived benefits and suitability for their needs, regardless of their regulatory status.

Additionally, we have only included German-language comments in our analysis (which represent a total of ~90% of collected reviews). This limits our findings to German-speaking populations only.

Another limitation is the difficulty to control for confounding factors, as there may be many factors other than the app itself that influence the user’s rating and review content (such as e.g., public discourse or opinions of authority figures such as health practitioners). While the study can shed some light on user-perceived therapy effectiveness, it is not able to establish a causal relationship between app usage and health outcomes, which limits the understanding of the relationship between the two. The meaningfulness of user-generated content (ratings and reviews) in the evaluation of mHealth applications is a key concern that merits further exploration, particularly regarding the correlation with patient outcomes and the reflection of patients’ and physicians’ priorities. This study constitutes a crucial component in the ongoing efforts of the digital medicine community to develop digital medical products that are effective and prioritize the needs of patients, however, large knowledge gaps remain.

Finally, the number of DiGAs in the directory is growing constantly (to over 40 in March 2023), meaning our sample represents only a small share of DiGAs consumers face (and will likely see in the future). However, considering the novelty of the topic and the number of reviews and apps included for analysis, we believe our study provides valuable insights and learnings from the unique German system of reimbursement and prescription of mHealth apps—and offers ample potential to replicate the results at a later point in time. Further research into the development of user experience over time, particularly user-perceived therapy effectiveness, would be interesting. It would also be worthwhile to investigate price-value and the economic effect of these apps on the German health insurance system. To advance the field of mHealth, future research should also investigate the validity and utility of patient-generated ratings as a measure of treatment effectiveness and explore their association with clinically meaningful outcomes and patient-centered endpoints.

The research on the effect of prescription and reimbursement on mHealth app user experience is still in its infancy. However, this study provides valuable insights into the user experience of mHealth apps under the unique mHealth prescription and full reimbursement system in Germany. It highlights not only that DiGAs have higher star ratings when comparing the same timeframe to comparators, but also that there are significant differences in user experience as derived by main topics. While users comment positively on customer service, personalization options, and reimbursement status for DiGAs, they reflect negatively on basic functionality and data input. In contrast, the comparator group receives more generic positive feedback with a majority of positive reviews falling into the category “generic laudatory talk”, and negative comments centering around pricing.

This study pioneers the usage of BERTopic in mHealth and highlights the importance of considering the user experience in mHealth app development and implementation. The results indicate that DiGAs, as a concept, are generally well-liked by users. However, it is crucial to maintain controls to limit the potential for excessive pricing and ensure consistent value-add even after clinical trials have been concluded. The results highlight the existing problem with consumer apps around high pricing and scam tactics that come with a lowly-regulated mHealth marketplace.

Regulators could consider making user experience an integral part of the review process for mHealth apps, as it is core to adoption and adherence. Additionally, given that positive user comments on therapy effectiveness are equally frequent between DiGAs and the comparator group in spite of the comprehensive DiGA certification process, good communication of therapy effectiveness is also key and should come from practitioners, general information campaigns by regulators or health insurance companies as well as providers.

## Methods

### Overview

This study uses a mixed-methods approach, which involves both quantitative and qualitative data collection and analysis. First, we conduct a comparison of average user star ratings using the Mann–Whitney–Wilcoxon test to contrast between DiGAs and comparators. In the second step, we perform a sentiment analysis with BERT (Bidirectional Encoder Representations from Transformers) as well as topic modeling with the novel BERTopic model in Python to compare user sentiment and key topics in written reviews. Our study pioneers the application of BERTopic in mHealth and provides proof-of-concept for its viability and usefulness in this research domain. The purpose of our study is to answer the following research questions:

1. Does prescription and reimbursement status have a positive relationship with user experience as measured by average app store star ratings (in both the Apple App Store and the Google Play Store)?
2. Does prescription and reimbursement status lead to different user experiences as evaluated by main topics and sentiment analysis of written reviews? Are the major user concerns or complaints similar across both app groups?

As previous research shows^40–42^, there is often a discrepancy between an app’s numeric rating and the information expressed in the textual review, making an analysis of both necessary to fully understand user experience. A combination of analyses of numeric star ratings and textual reviews is required to provide a full and complete answer to the above-defined research questions.

### General app inclusion criteria and comparator selection

Since the aim of this work is to evaluate the relationship between the prescription and reimbursement status of mHealth apps and the user experience and contrast it against mHealth apps without reimbursement and prescription status, two groups were formed. The focus group consists of 15 DiGAs (mobile health apps with reimbursement and prescription status in Germany), as listed in the official DiGA directory in August 2022. This represents all DiGAs listed in the directory for a period of at least 12 months at that point in time. All DiGAs are cross-platform apps, meaning they are available in both app stores. According to a report on aggregated sales figures^25^, Android and iOS phones account for 99% of the German mobile operating system market, covering almost all German smartphone users. To ensure the quality of statistical analyses, only apps with at least 50 total ratings (iOS and Android ratings added together) were included. The identified DiGAs target 11 different indications, as identified by their corresponding ICD codes (for more detail, see Table 1). The inclusion in the sample of two mHealth apps that retracted their application midway through the observation period (M-Sense, 04/04/2022, and MIKA; 27/03/2022) may be perceived as a limitation. However, it is important to note that these two apps represent only a small portion of the total sample, accounting for 2 out of 15 apps. Moreover, both apps were certified during most of the observed period and lost their certification only toward the end of the period.

**Table 1:** Overview of selected DiGAs and corresponding ratings and reviews.

Therapeutic area	Indication	# Comparators	DiGA name	Status	Date of entry into DiGA directory	Included in statistical analyses	Android: # of downloads	Android: # ratings	Android: average star ratings	Android: AUR	Android: # textual reviews	iOS: # ratings	iOS: average star ratings	iOS: AUR	iOS: # textual reviews	Overall: total # ratings	Overall: # reviews prior to entry into DiGA directory
*Diseases of the cardiovascular system*	*Stroke/cerebral infarction*		*Rehappy*	*Preliminary*	*12/29/2020*	*No*	*>500*	*5*	*4.8*			*7*	*3.9*			*12*	*1*
Diseases of the digestive system	Irritable bowel syndrome	4	Cara Care	Preliminary	12/26/2021	Yes	>10,000	194	3.2	4.21	68	301	4.5	4.41	40	495	83
Diseases of the ear	Tinnitus aurium	4	Kalmeda	Permanent	10/6/2020	Yes	>10,000	216	2.4	4.21	154	84	3.1	4.39	58	300	41
			*Meine Tinnitus App*	*Preliminary*	*03/06/2022*	*No*	*>500*	*22*	*4.5*			*8*	*4*			*30*	
Diseases of the genitourinary system	Impotence of organic origin	3	Kranus Edera	Preliminary	12/18/2021	Yes	>1000	40	4.4	4.22	17	54	4.5	4.41	12	94	0
Diseases of the musculoskeletal system	Back pain	6	Vivira	Permanent	10/22/2020	Yes	>10,000	311	4.4	4.22	160	504	4.7	4.43	81	815	0
Diseases of the nervous system	Migraine	4	M-sense Migräne	Retracted	12/16/2020	Yes	>10,000	1160	3.8	4.21	123	276	4.7	4.42	26	1436	113
Endocrine, nutritional, and metabolic diseases	Diabetes	7	ESYSTA	Preliminary	07/04/2021	Yes	>10,000	82	3.8	4.22	24	8	3.4	4.41	4	90	21
			Vitadio	Preliminary	04/15/2022	Yes	>1000	87	4	4.22	8	6	4.7	4.41	3	93	24
	Obesity	8	Oviva Direkt	Preliminary	10/3/2021	Yes	>1000	45	4.5	4.22	13	33	4.3	4.41	3	78	0
			zanadio	Preliminary	10/22/2020	Yes	>50,000	1010	4.7	4.23	740	341	4.3	4.4	120	1351	0
Mental and behavioral disorders	Non-organic insomnia	10	somnio	Permanent	10/22/2020	Yes	>10,000	330	4.2	4.22	115	365	4.4	4.41	45	695	0
	Social phobias & agoraphobia, panic disorder	5	Invirto	Preliminary	12/3/2020	Yes	>5000	30	4.1	4.22	21	52	4.1	4.4	13	82	0
			Mindable	Preliminary	04/29/2021	Yes	>1000	31	4.1	4.22	13	30	4.2	4.41	6	61	7
	Tobacco: dependence syndrome	8	Nichtraucher Helden	Preliminary	07/03/2021	Yes	>100,000	571	4.3	4.22	210	185	4.6	4.41	16	756	8
Oncology	Cancer	1	CANKADO	Preliminary	05/03/2021	Yes	>1000	48	3.9	4.22	4	10	3.6	4.41	1	58	2
			Mika	Retracted	03/25/2021	Yes	>100,000	141	4.1	4.22	56	39	4.1	4.41	13	180	44
			*PINK! Coach*	*Preliminary*	*06/27/2022*	*No*	*>1000*	*6*	*2.8*			*3*	*3.7*			*9*	*5*
*Other*	*Dysphasia & aphasia, apraxia*		*neolexon Aphasie*	*Preliminary*	*02/06/2022*	*No*	*>5000*	*10*	*5*			*3*	*4.3*				*4*

*Italic formatting* = excluded from statistical analyses.

Status = refers to whether an app has been included in the DiGA directory permanently (which means clinical proof has been submitted, reviewed, and accepted) or preliminary (with clinical proof outstanding).

AUR = refers to “aggregate user-perceived rating” (as defined in Equation (1).

The lack of medical evidence for most apps (given their provisional approval status) could be seen as another constraint, yet it accurately reflects the reality consumers face under the DiGA system. Furthermore, unlike the comparator group, the DiGAs were all subject to an initial benefit assessment by BfArM. Another aspect worth mentioning is the fact that several DiGAs (10/15) were already on the market before the introduction of the DVG and thus before inclusion in the DiGA registry. Since it is not possible to separate the star ratings by time period, the analysis of the ratings includes some data from this period. However, if we consider the number of textual reviews as a proxy, the total percentage of such reviews amounts to only 16.7%. Furthermore, it could be argued that the overall app content and value proposition are unlikely to have changed significantly between the time these apps entered the market and the launch of the DiGA directory.

**Fig. 7:**
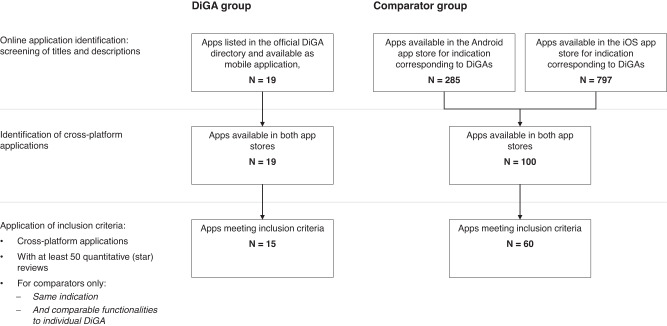
Flow diagram for smartphone app selection. The “DiGA group” represents all official DiGAs. The “Comparator group” describes corresponding health apps. Both had to meet the inclusion criterias mentioned.

The comparator group consists of 60 consumer mHealth apps without a prescription and reimbursement status. To identify appropriate comparators, we first conducted a broad search in both German app stores (Android and iOS). We performed a keyword search based on the 11 indication areas targeted by the DiGAs (see indication column in Table 1), yielding 797 applications in the iOS and 285 applications in the Android app store. These apps comprise all types of consumer apps, including diet trackers and fitness apps. Next, cross-platform apps were identified, rendering 100 remaining apps. In the last step, we manually reviewed all apps and applied inclusion criteria, only including applications that had comparable claimed functionalities (as defined in the app description and screenshots) to the individual DiGA within their indications and possessed at least 50 total ratings, leading to a comparator group size of 60 apps (for more detail on app selection, see Fig. 7). Additionally, Tinnitracks, an app for tinnitus patients, was removed from the comparator group as it is widely reimbursed by insurances through selective agreements despite not having DiGA status and could thus cause bias in the analyses.

### Data collection

All steps of this research project were approved by the Ethics Committee of Witten/Herdecke University (Nr. S-288/2021). For the empirical analyses, two different datasets were used. The first dataset was compiled manually and included basic app data such as app ID, name, category, average user star rating, and the number of ratings. The second dataset focused on textual review data and collected information such as review title, full review text, username, star rating, and developer response. The textual review data from both app stores were collected using open-source scrapers for Python 3.11., namely Google Play Store Scraper^43^ and Apple Store Scraper^44^. All data were stored on the local server according to the university’s research data management guidelines. Textual reviews were collected from the first date of entry of the relevant indication into the DiGA directory—August 4, 2022, resulting in a total of 17,588 German language reviews, including 2080 DiGA reviews and 15,508 comparator reviews.

### Empirical analyses: comparing differences between groups—average user star ratings

The average user star rating is calculated automatically by the platform provider based on all app ratings, including those without a textual review. Comparing highly rated apps with few ratings to those with a lower star rating but a high number of user ratings can be challenging^45,46^. To address this, we computed an aggregate user-perceived rating (AUR) per app as suggested by Joorabchi^20^, which considers both the star rating and the number of ratings. Due to the high spread of ratings in our dataset (min = 50, max = 546,000, SD = 109,669), we adapted the formula to use medians instead of averages as follows:

Equation (1): Aggregate user-perceived rating (AUR)

1 $${AUR}\left({{app}}_{i}\right)=\frac{{v}_{i}\times {r}_{i}}{{v}_{i}+m}+\frac{m\times c}{{v}_{i}+m}$$

Where:${v}_{i}$ is the number of ratings for ${{app}}_{i}$, ${r}_{i}$ is the average star rating for the ${{app}}_{i}$, $m$ is the median number of ratings (for all apps in the dataset), $c$ is the median star rating (for all apps in the dataset)

The comparison of star ratings between groups was then performed using R 4.1.3. As the star rating data does not follow a normal distribution (proven by Q-Q-plot), the non-parametric Mann–Whitney–Wilcoxon^47,48^, test was employed to compare rating distribution. To ensure comparability of medians, we tested for homogeneity of variances using Levene’s^49^ test. In order to quantify the difference in star ratings, we calculate the power of the effect according to Cohen^16^.

### Empirical analyses: comparing user experience as evaluated by main topics and sentiment analysis of written reviews

Text mining was conducted using Python 3.11, using textblob^50^ in conjunction with the German sentiment classifier BERT^51^ for sentiment analysis and BERTopic^52^, a form of unsupervised machine learning, for topic modeling.

Data pre-processing steps included transforming text to lowercase, removing punctuation, stopwords and emoticons, lemmatizing text, and tokenizing remaining text. Sentiment analysis was performed using a lexicon and rule-based approach, assigning polarity scores ranging from −1 to 1 using the NLTK library^53^. Latent Dirichlet Allocation (LDA)^54^ was first used for topic modeling, but the more novel BERTopic^55^ was found to be a better fit due to its ability to account for contextual semantics and grammatical roles of words. Because the model achieves higher accuracy rates (= share of reviews allocated to subjectively reasonable categories) with less contradictory statements, reviews were first split into positive statements (a star rating of 4 and 5) and criticism (a star rating of 1 or 2). In the second step, 10 iterations of the model were run to identify the main topics per app group and review type. Outliers were excluded from further analysis. The resulting topics were manually coded by three researchers and assigned to overarching categories using Grounded Theory^56^ in an open coding, blind approach, with inter-rater agreement measured as strong (kappa = 0.83)^57^. The allocations from all individual runs were then aggregated to form an average percentage of reviews assigned to each overarching category.

### Reporting summary

Further information on research design is available in the Nature Research Reporting Summary linked to this article.

## Supplementary information

Supplementary material

Reporting Summary

## Data Availability

The data that support the findings of this study are available from the corresponding author upon reasonable request.

## Code availability

The code that supports the findings of this study is available from the corresponding author upon reasonable request.
