# Supplementary material for: Rating analysis and BERTopic modeling of consumer versus regulated mHealth app reviews in Germany
Source: NPJ Digit Med. 2023 Jun 21;6:115. doi: 10.1038/s41746-023-00862-3 (PMC10285024; doi:10.1038/s41746-023-00862-3)
Supplement: Supplementary file 1 — Supplementary material [file 41746_2023_862_MOESM1_ESM.pdf]

Supplementary material for “User experience with consumer versus regulated mHealth apps in Germany: a rating analysis and BERTopic-based topic modeling of app store reviews”

Supplementary notes: Secondary results

Apps on the Android platform have 5.5 times as many ratings as on iOS

Looking into the basic app data collected, the apps on average had 48,792 total ratings across platforms (median = 2,900; SD = 134,573). Due to their recent introduction and thus shorter time on the market, DiGAs have a significantly lower number of ratings compared to the comparator group: on average, 439 users rated a DiGA (vs. 61,085 for a comparator), with a median of 180 ratings (vs. 5,647).

Comparing platforms against each other, applications in the Google Play Store have, on average, almost 5.5x as many user ratings as on the Apple App Store (Google Play Store average = 40,946 ratings, median = 1,901 vs. Apple Play Store average = 7,316 ratings, median = 120).

Looking into indications, obesity, insomnia, and social phobias / panic disorders seem to be the better-established indications with the highest number of average user ratings (174,290, 105,910 and 30,023 average ratings respectively), whereas apps targeting cancer and impotence have the lowest average number of ratings (96 and 340).

Apps on iOS are, on average, rated 0.19 stars higher (median = 0.3 stars higher)

While users tend to rate the selected apps on Android more, apps on iOS have a higher average star rating (average iOS = 4.41 stars, median = 4.60 vs. average Android = 4.22 stars, median = 4.30; difference between groups is significant at $p < 0.01$; $z = -3.16$; $r = 0.36$; non-parametric Mann-Whitney-Wilcoxon test). Based on Cohen [1], the power of this effect is medium. The same holds true when looking into the aggregated user-perceived rating (AUR): for apps on the iOS platform, the AUR is 4.43, while the AUR on Android is 4.25 ($p < 0.001$; $z = -6.76$; $r = 0.78$; non-parametric Mann-Whitney-Wilcoxon test); here, the effect can be classified as large. Out of our selected sample of 75 apps, 56 (75%) do not have consistent star rating across platforms (as defined by a higher than 0.1-star variation up- or downward).

Supplementary Table 1: Topic modeling category definition

Type	Category code	Definition

**DiGA positive**	Therapy area specific	Reviews that contain keywords very specific to the relevant therapy area (e.g., adipositas; review highlighting " I lost 3 **kg** already" or "[…] great **recipes**!") automatically get allocated by the model into one category. As the reviews allocated to this category contain a mixture of topics (ranging from e.g., laudatory talk to ease of use to therapy effectiveness), this category offers little informational content for our analyses.
	Customer service & personalization	Reviews praising availability, quality and positive attitude of customer service as well as the options to personalize the app, either through specific settings or jointly with customer service, fall into this category.
	Ease of use	Reviews highlighting ease of use and low complexity of setting up the app fall into this category.
	Generic laudatory talk	Reviews in this category are mostly short and include laudatory, but unspecific remarks such as, e.g., "**Fantastic** app", "I definitely **recommend** this app […]".
	Therapy effectiveness	Reviews in this category contain phrases and words related to the general helpfulness, impact and postive effect of the app on the user's health and well-being.
	Motivational effect	Reviews that center around positive attitude change and contain keywords such as e.g., "**motivated**" "**excited** to do exercises" fall within this category.
	Availability of specific functions or content formats (e.g., video)	Reviews in this category praise the availability of specific functions and content formats. They contain keywords such as, e.g., "**video**", "**audio**", "**clip**", in connection with positive adjectives and/ or laudatory talk.
	Reimbursement status and activation process	Reviews in this category contain phrases and words related to **reimbursement** (i.e., complimenting, expressing gratitude/ positive feeling related to reimbursement status, such as e.g., "[...] amazing that the health insurance covers this app! [...]"), **activation (codes)**, **(statutory) health insurance** in connection with positive adjectives and/ or laudatory talk.
	Content quality	Reviews in this category compliment the quality of the content and contain keywords such as, e.g., "**informative**", "**informational content**", "**reliable information**"
	Wish for improved connectivity of devices	Reviews in this category contain laudatory talk and positive adjectives in combination with keywords around device connectivity, such as e.g., "**(fitness-)tracker**" or "**bluetooth**" and expressions of desire, such as e.g., "I **wish**", "**would be** great", "[...] **could**".
	Data input	Reviews in this category contain keywords around data input, such as e.g., "**entry**", "**enter**", "**input**", "**query**", in connection with positive adjectives and/ or laudatory talk.
DiGA negative	Basic functionality: bugs/ crashes	Reviews in this category complain about aspects of basic functionality, such as e.g., frequent app crashes, bugs, and blank loading screens.
	Login/ Registration	Reviews in this contain keywords around either **login** or **registration**, in connection with negative adjectives and critical/ uncomplimentary talk.
	Therapy area specific	Reviews that contain keywords very specific to the relevant therapy area (e.g., adipositas; review highlighting " I lost 3 **kg** already" or "[…] great **recipes**!") automatically get allocated by the model into one category. As the reviews allocated to this category contain a mixture of topics (ranging from e.g., data input, limited value add or pricing), this category offers little informational content for our analyses.
	Data input	Reviews in this category contain keywords around data input, such as e.g., "entry", "enter", "input", "query", in connection with negative adjectives and critical/ uncomplimentary talk.
	Limited value add	Reviews in this category contain three different sets of keywords: the first centers around negations in connection with different variations of general helpfulness, impact and postive effect of the app on the user's health and well-being (e.g., "**Not helpful**", "**no effect** on my health", "no **difference**"). The second revolves around mentions of freely available offers that have the same or higher benefit (e.g., "**youtube videos** are more effective" or "[...] **no different** than the notes app on my phone"). The last contains reviews questioning the purpose and/ or benefits of the app (e.g., "[...] **wondering** about the **purpose** [...]").
	Complaints about reimbursement status and process in lieu of limited value add	Reviews in this category contain one of the three different types of keywords mentioned under "limited value add" in connection with keywords around reimbursement (status) and/ or (statutory) health insurance.

	Issues with specific functions or content formats (e.g., video)	Reviews in this category focus on specific functions and content formats. They contain keywords such as, e.g., "**video**", "**audio**", "**clip**", in connection with negative adjectives and critical/ uncomplimentary talk.
	Pricing	Reviews in this category complain about (excessive) pricing, containing keywords such as .e.g., "**price**", "**pricing**", "**cost**", in connection with adjectives expressing displeasure about the pricing model and the user's opinion that prices are too high (such as e.g., "**excessive**", "**too expensive**", "**unbelievably high**"). The difference between this category and the category "Complaints about reimbursement status and process in lieu of limited value add" is that the reviews in this category do not mention any of the keywords around limited value add.
	Reimbursement status and activation process	Reviews in this category contain phrases and words related to reimbursement (e.g., "[…] **covered** by the health insurance"), activation (codes), (statutory) health insurance in connection with negative adjectives and critical/ uncomplimentary talk.
Comparator positive	Therapy area specific	Reviews that contain keywords very specific to the relevant therapy area (e.g., adipositas; review highlighting " I lost 3 **kg** already" or "[…] great **recipes**!") automatically get allocated by the model into one category. As the reviews allocated to this category contain a mixture of topics (ranging from e.g., laudatory talk to ease of use to therapy effectiveness), this category offers little informational content for our analyses.
	Generic laudatory talk	Reviews in this category are mostly short and include laudatory, but unspecific remarks such as, e.g., "**Fantastic** app", "I definitely **recommend** this app […]".
	Ease of use	Reviews highlighting ease of use and low complexity of setting up the app fall into this category.
	Therapy effectiveness	Reviews in this category contain phrases and words related to the general helpfulness, impact and postive effect of the app on the user's health and well-being.
	Laudatory talk and issues with pricing	Reviews in this category include laudatory, but unspecific remarks such as, e.g., "**Fantastic** app", "I definitely **recommend** this app […]" combined with complaints about the pricing model. Relevant keywords would include, e.g., "price", "pricing", "cost", in connection with adjectives expressing displeasure about the pricing model and the user's opinion that prices are too high (such as e.g., "excessive", "too expensive", "unbelievably high")
	Laudatory talk and issues with fuctionality	Reviews in this category include laudatory, but unspecific remarks such as, e.g., "**Fantastic** app", "I definitely **recommend** this app […]" combined with complaints about functionality, such as e.g., frequent app crashes, bugs, and blank loading screens.
	Improved monitoring/ self-control	Reviews in this category compliment the user's better ability to **self-monitor** and exercise **self-control**.
	Motivational effect	Reviews that center around positive attitude change and contain keywords such as e.g., "**motivated**" "**excited** to do exercises" fall within this category.
	Laudatory talk and language issues	Reviews in this category include laudatory, but unspecific remarks such as, e.g., "**Fantastic** app", "I definitely **recommend** this app […]" combined with complaints about language issues, mainly the lack of availability of the app or specific app content in German language.
	Content quality	Reviews in this category compliment the quality of the content and contain keywords such as, e.g., "**informative**", "**informational content**", "**reliable information**"
	Laudatory talk and issues with ads	Reviews in this category include laudatory, but unspecific remarks such as, e.g., "**Fantastic** app", "I definitely **recommend** this app […]" combined with complaints about frequent ads.
	Availability of specific functions or content formats (e.g., video)	Reviews in this category praise the availability of specific functions and content formats. They contain keywords such as, e.g., "**video**", "**audio**", "**clip**", in connection with positive adjectives and/ or laudatory talk.
	Improved relationship with physician	Reviews in this category highlight the user's improved raltionship and/or communication with their physician.
Comparator negative	Pricing	Reviews in this category complain about (excessive) pricing, containing keywords such as .e.g., "**price**", "**pricing**", "**cost**", in connection with adjectives expressing displeasure about the pricing model and the user's opinion that prices are too high (such as e.g., "**excessive**", "**too expensive**", "**unbelievably high**").

Therapy area specific	Reviews that contain keywords very specific to the relevant therapy area (e.g., adipositas; review highlighting " I lost 3 **kg** already" or "[…] great **recipes**!") automatically get allocated by the model into one category. As the reviews allocated to this category contain a mixture of topics (ranging from e.g., data input, limited value add or pricing), this category offers little informational content for our analyses.
Issues with or after updates	Reviews in this category complain about issuews with functionality, such as e.g., frequent app crashes, bugs, and blank loading screens, in connection with keywords around "**updates**".
Limited value add	Reviews in this category contain three different sets of keywords: the first centers around negations in connection with different variations of general helpfulness, impact and postive effect of the app on the user's health and well-being (e.g., "**Not helpful**", "**no effect** on my health", "no **difference**"). The second revolves around mentions of freely available offers that have the same or higher benefit (e.g., "**youtube videos** are more effective" or "[...] **no different** than the notes app on my phone"). The last contains reviews questioning the purpose and/ or benefits of the app (e.g., "[...] **wondering** about the **purpose** [...]").
Basic functionality: bugs/ crashes	Reviews in this category complain about aspects of basic functionality, such as e.g., frequent app crashes, bugs, and blank loading screens.
Issues with specific functions or content formats (e.g., video)	Reviews in this category focus on specific functions and content formats. They contain keywords such as, e.g., "**video**", "**audio**", "**clip**", in connection with negative adjectives and critical/ uncomplimentary talk.
Scam/ rip-off	Reviews in this category complain about (excessive) pricing combined with scam/ rip-off schemes, containing keywords such as .e.g., "**price**", "**pricing**", "**cost**", in connection with "**rip-off**", "**scam**", and warnings to other users, such as .e.g., "**careful**", "**beware**".
Language issues	Reviews in this category complain about language issues, mainly the lack of availability of the app or specific app content in German language.
Ads	Reviews in this category complain about frequent ads.
No ease of use	Reviews highlighting a lack of ease of use and high complexity of setting up the app fall into this category.
Issues with account cancellation	Reviews in this contain keywords around account/ subscription cancellation, in connection with negative adjectives and critical/ uncomplimentary talk.
Login/ registration	Reviews in this contain keywords around either **login** or **registration**, in connection with negative adjectives and critical/ uncomplimentary talk.
Data security	Reviews expressing concerns with data protection and data security fall into this category.
Data input	Reviews in this category contain keywords around data input, such as e.g., "entry", "enter", "input", "query", in connection with negative adjectives and critical/ uncomplimentary talk.

Supplementary References

[1] J. Cohen, *Statistical Power Analysis for the Behavioral Sciences by Jacob Cohen (1988-08-12)*, 2nd ed. Routledge, 2013.
